# Validation of a color deconvolution method to quantify MSC tri-lineage differentiation across species

**DOI:** 10.3389/fvets.2022.987045

**Published:** 2022-10-13

**Authors:** Emma Heyman, Marguerite Meeremans, Bert Devriendt, Maria Olenic, Koen Chiers, Catharina De Schauwer

**Affiliations:** ^1^Veterinary Stem Cell Research Unit, Department of Translational Physiology, Infectiology and Public Health, Faculty of Veterinary Medicine, Ghent University, Merelbeke, Belgium; ^2^Laboratory of Immunology, Department of Translational Physiology, Infectiology and Public Health, Faculty of Veterinary Medicine, Ghent University, Merelbeke, Belgium; ^3^Tissue Engineering Lab, Muscles and Movement Group, Faculty of Medicine, Catholic University of Leuven, Kortrijk, Belgium; ^4^Laboratory of Veterinary Pathology, Department of Pathobiology, Pharmacology and Zoological Medicine, Faculty of Veterinary Medicine, Ghent University, Merelbeke, Belgium

**Keywords:** tri-lineage differentiation, quantification, regenerative medicine, donor variability, MSC

## Abstract

Mesenchymal stem cells (MSCs) are a promising candidate for both human and veterinary regenerative medicine applications because of their abundance and ability to differentiate into several lineages. Mesenchymal stem cells are however a heterogeneous cell population and as such, it is imperative that they are unequivocally characterized to acquire reproducible results in clinical trials. Although the tri-lineage differentiation potential of MSCs is reported in most veterinary studies, a qualitative evaluation of representative histological images does not always unambiguously confirm tri-lineage differentiation. Moreover, potential differences in differentiation capacity are not identified. Therefore, quantification of tri-lineage differentiation would greatly enhance proper characterization of MSCs. In this study, a method to quantify the tri-lineage differentiation potential of MSCs is described using digital image analysis, based on the color deconvolution plug-in (ImageJ). Mesenchymal stem cells from three species, i.e., bovine, equine, and porcine, were differentiated toward adipocytes, chondrocytes, and osteocytes. Subsequently, differentiated MSCs were stained with Oil Red O, Alcian Blue, and Alizarin Red S, respectively. Next, a differentiation ratio (DR) was obtained by dividing the area % of the differentiation signal by the area % of the nuclear signal. Although MSCs isolated from all donors in all species were capable of tri-lineage differentiation, differences were demonstrated between donors using this quantitative DR. Our straightforward, simple but robust method represents an elegant approach to determine the degree of MSC tri-lineage differentiation across species. As such, differences in differentiation potential within the heterogeneous MSC population and between different MSC sources can easily be identified, which will support further optimization of regenerative therapies.

## Introduction

The therapeutic potential of mesenchymal stem cells (MSCs) has received much attention in regenerative medicine based on their ability to proliferate, differentiate toward various lineages and modulate immune responses (1, 2). This is illustrated by the numerous clinical trials which are currently performed to evaluate MSC-based therapies for the treatment of both musculoskeletal and non-musculoskeletal diseases, ranging from tendon lesions and osteoarthritis to neurological and cardiac abnormalities (3).

As stated by the International Society for Cellular Therapy (ISCT), human MSCs are characterized using following criteria: (i) morphological features (plastic-adherency), (ii) presence of MSC markers (CD73, CD90, and CD105) and lack of hematopoietic markers (CD11b or CD14, CD34, CD45, CD79α or CD19 and MHC class II), and (iii) tri-lineage differentiation toward adipo-, chondro- and osteocytes (4). However, a proper characterization of MSCs is hampered by the heterogeneity within the MSC population and the presence of species-specific differences in veterinary medicine, with variations in proliferation potential, differentiation capacity, and protein expression profile (2, 3, 5). Therefore, additional guidelines were suggested in a recent position statement by Guest et al. to be incorporated in the minimal definition criteria of MSCs, including *in vitro* immune suppression assays and quantitative assessment of tri-lineage differentiation (3). Indeed, MSCs should be unequivocally characterized to ensure that reproducible MSC-products are used in veterinary clinical trials which will yield useful and clinically relevant outputs (3).

Tri-lineage differentiation ability of MSCs is commonly assessed based on qualitative analysis of histological stainings, such as Oil Red O staining for adipogenic differentiation, Alcian Blue or Safranin O staining for chondrogenic differentiation and Alizarin Red S or Von Kossa staining for osteogenic differentiation (6–11). Visual identification and histological scoring of tri-lineage differentiation remains however subjective (Table 1). Furthermore, a qualitative evaluation of representative histological images does not always unambiguously confirm tri-lineage differentiation. Moreover, potential differences in differentiation capacity between different donors or sources are not identified. Therefore, quantitative assays for tri-lineage differentiation should be optimized and implemented.

**Table 1:** An overview of the advantages and disadvantages of techniques mostly used for quantification of MSC tri-lineage differentiation.

	**Advantages**	**Disadvantages**
Histological scoring (BF and HC)	Short assay time Limited number of cells required Straightforward and inexpensive	Highly subjective Qualitative and semiquantitative
Absorbance (Spectrophotometry)	Short assay time Straightforward	Destructive Low sensitivity and selectivity Effect of sample conditions (pH, T°) and device settings Variation (degradation of organic solvent) Cell-to-cell heterogeneity and cell numbers not identified Qualitative and semiquantitative
Fluorescence and IHC	High sensitivity Wide range of fluorophores Diverse readout modes Less subjective	Validation/lack of species-specific Abs Time consuming Autofluorescence Non-specific binding of Abs Photobleaching Interference of Abs (multi-color) Qualitative and semiquantitative
Flow cytometry	Short assay time Highly quantitative at protein level Identification of cell number, size, and granularity Less subjective	High number of cells required (10^5^ cells/test) Cells should be detached (in suspension) Need for accurate gating strategy Validation/lack of species-specific Abs Underestimation of cell numbers (due to washing and detaching)
Western blotting	Sensitive and reproducible Straightforward	Time consuming Cell lysis for protein isolation Cell-to-cell heterogeneity not identified Qualitative and semiquantitative
RT-qPCR	Sensitive and reproducible Quantitative at gene expression level	Destructive (RNA extraction) No validation at the translational level Primer validation/choice of reference genes Time consuming Cell-to-cell heterogeneity not identified

Abs, antibodies; BF, brightfield; HC, histochemistry; IHC, immunohistochemistry; RT-qPCR, reverse transcription quantitative polymerase chain reaction.

A first method to quantify tri-lineage differentiation includes spectrophotometric measurements based on the elution of different histological staining solutions using volatile organic solvents such as isopropanol (12–15). After staining, the dye is eluted and the amount of light absorbed by the eluate is measured, hence the short assay time for absorbance assays. Absorbance assays, however, are less appropriate for samples with low signal as UV-Vis spectrophotometry has a low sensitivity. Moreover, the selectivity of these assays might be limited as contaminants like nucleic acids might absorb at the same wavelength. Results might be affected as well by different sample conditions such as pH and temperature, and device settings are of major importance (e.g. calibration) (16). Additionally, the absorbance of samples is subjected to variation caused by the degradation of isopropanol over time and information on MSC heterogeneity and cell numbers is lacking (Table 1) (17).

Besides histological stainings, fluorescence-based techniques are used to quantify tri-lineage differentiation, which are known for their high sensitivity, wide range of fluorophores and various readout modes such as fluorometry, fluorescent image analysis and flow cytometry (18). The expression and concentration of certain proteins can be measured based on total intensity of a fluorescent label. To quantify adipogenic differentiation, the expression of following proteins can be evaluated: CAAT/enhancer binding protein a (C/EBPa), fatty acid binding protein (FABP) 4, insulin-like growth factor binding protein 2 (IGFBP2), resistin, adiponectin and lipoprotein lipase. Expression of osteoblast-related transcription factors and proteins include osteocalcin, osteopontin, Runt-related transcription factor 2 (Runx2), alkaline phosphatase and collagen type 1 (19), while SRY-Box Transcription Factor 9 (SOX9) and aggrecan (ACAN) are expressed during chondrogenic differentiation (20). Although fluorometry is superior to spectrophotometry in terms of sensitivity and specificity, limitations related to the elution of specific components remain (21). Alternatively, fluorescent stainings using antibodies can be performed, although their use in veterinary medicine is hampered by the lack of species-specific or validated cross-reactive antibodies (13, 22). Fluorescence assays are also more time-consuming and more complicated to perform due to potential autofluorescence, non-specific binding of antibodies, photobleaching and interference due to spectral overlap when multiple antibodies are used (Table 1) (23). To evaluate adipogenic differentiation of MSCs, specific lipophilic fluorescent stainings which do not require antibodies, such as Nile Red or AdipoRed, are being used as well. The intensity of the staining can be measured and compared against 4′,6-diamidino-2-phenylindoline (DAPI) nuclear staining (17, 24). However, similar stainings are not available for chondrogenic and osteogenic differentiation.

Other protein expression analysis techniques such as flow cytometry and western blotting can be used to quantify tri-lineage differentiation. So far, relatively few studies used flow cytometry to confirm MSC tri-lineage differentiation, although a lot of information would be provided, such as intensity of staining, size of the cells, granularity, as well as cell number and MSC heterogeneity (25). However, a high number of cells is required (10^5^ cells/test), when compared with other methods and the cells should be detached prior to analysis. Additional technical challenges include the validation of antibodies and underestimation of the number of differentiated cells due to cell loss after the washing steps or trypsinization prior to analysis (Table 1) (22). Western blotting is a semiquantitative method based on the immunochromatography principle. Although it is commonly used because of its high sensitivity and reproducibility, it is time consuming, additionally requires cell lysis for protein isolation and does not give information about MSC heterogeneity (Table 1) (26).

As species-specific and validated cross-reacting antibodies are often lacking, a third method for quantification is based on gene expression analysis techniques, such as reverse transcription quantitative PCR (RT-qPCR). This sensitive and reproducible tool is based on the amplification of specific differentiation-related genes compared to the expression of reference genes (12, 27). However, RT-qPCR only reflects changes at the gene expression level without any confirmation on the translation level, taking into account that mRNA levels are not always directly correlated with protein levels (28). Furthermore, attention should be paid to the correct selection of reference gene(s) and primer validation of the genes of interest when designing RT-qPCR protocols (17, 29). Other disadvantages of this technique are the long assay time and the fact that MSC heterogeneity cannot be identified using RT-qPCR (Table 1).

In human research, quantification of MSC differentiation is frequently reported, however, techniques are inconsistent between research groups and often specialized equipment is needed such as imaging and absorbance readers (15, 17, 30–32). Furthermore, the used methodology or programs, for example ImageJ macro's, are not always clearly described nor available for future research (31). In veterinary studies, only few studies describe quantification of MSC differentiation and even then a complete quantification of each of the three lineages, accompanied by a clearly described method, is often lacking (13, 27, 33, 34). For example, Jurek et al. quantitatively assessed adipogenic differentiation capacity of bovine MSCs by spectrophotometric analysis of Nile red/DAPI staining, while osteogenic and chondrogenic differentiation were not quantified (27). The latter was also not reported in the study of Zimmermann et al. in which porcine MSCs were characterized (34). Usually, a proper negative control for chondrogenic differentiation is not included as well (35). Hillmann et al. clearly described the quantification of the three lineages, although different techniques were used in this study to quantify MSC differentiation, namely histological scoring to evaluate adipogenic and chondrogenic differentiation, while optical density measurements were used to evaluate osteogenic differentiation (13). The use of color deconvolution for quantification of one or two MSC differentiation lineages was described in some recent papers. For example, Hagen et al. used color deconvolution for quantification of chondrogenic differentiation (36) and Brandt et al. reported color deconvolution in Mathematica in combination with area determination for adipogenic and chondrogenic differentiation (37).

As far as we know, no universal quantification technique is available which can be used for all lineages. To enhance the implementation of tri-lineage differentiation quantification when characterizing MSCs, it is recommended to develop a single quantification technique that does not require additional handlings on top of the qualitative assessment which is currently routinely performed. To this end, a straightforward, simple but robust method for image-based quantification of tri-lineage differentiation using standard histological stainings was evaluated in this study in three different species i.e. bovine, equine and porcine.

## Materials and methods

### Media

Culture medium for undifferentiated MSCs consisted of low glucose Dulbecco's Modified Eagle Medium (DMEM-LG) (Invitrogen, 11880-036) supplemented with 30% Fetal Bovine Serum (FBS) (Sigma, F7524), 10^−11^ M dexamethasone (Sigma, D2915), 1% antibiotic-antimycotic solution (Sigma, A5955) and 1% L-glutamine (Invitrogen, 25030-024). Expansion medium was identical to the culture medium but without dexamethasone.

Following media were used to induce differentiation: (i) adipogenic induction medium consisting of DMEM-LG supplemented with 10^−6^ M dexamethasone, 0.5 mM 3-isobutyl-1-methylxanthine (Sigma, I7018), 10 μg/ml rh-insuline (Sigma, I9278), 0.2 mM indomethacin (Sigma, I7378), 15% rabbit serum (Sigma, R4505), 50 μg/ml gentamycin (Gibco, 15710-049) and 1% antibiotic-antimycotic solution; (ii) adipogenic maintenance medium, identical to the adipogenic induction medium except for the omission of dexamethasone, indomethacin and 3-isobutyl-1-methylxanthine; (iii) chondrogenic medium, based on the basal differentiation medium (Lonza, PT-3003), supplemented with 10 ng/ml Transforming Growth Factor-β3 (Lonza, PT-4124); and (iv) osteogenic medium, consisting of DMEM-LG supplemented with 10% FBS, 0.05 mM L-ascorbic acid-2-phosphate (Sigma, 49752), 10^−7^ M dexamethasone, 10 mM β-glycerophosphate (Sigma, G9422), 50 μg/ml gentamycin and 1% antibiotic-antimycotic solution (11).

### Cell isolation methods

Bovine and porcine MSCs were isolated from adipose tissue (eight and four donors, respectively), collected in the local abattoir, using enzymatic digestion. Briefly, adipose tissue was transported within 1–2 h to the lab in PBS containing 50 μg/ml gentamycin. After extensive washing with PBS to remove blood, tissue samples were cut in small pieces of approximately 1 mm^3^, transferred into a four-well plate, minced with a sterile pipet tip for 1–2 min in an enzymatic solution containing 1 mg/ml liberase (Sigma, LIBTM-RO), and incubated for 6 h at 38.5°C in a humidified atmosphere containing 5% CO_2_. Subsequently, the enzymatic reaction was neutralized with an equal amount of culture medium. The mixture was allowed to separate at room temperature (RT) and the non-buoyant fraction was collected over a 70 μm cell strainer. After adding an equal amount of pre-warmed culture medium, cells were centrifuged for 5 min at 400 g at RT. Finally, after two washing steps, all cells isolated from the 1 mm^3^ adipose tissue were suspended into culture medium, seeded in a 25 cm^2^ culture flask and cultured at 38.5°C in a humidified atmosphere containing 5% CO_2_. Non-adherent cells were removed the following day by completely replacing the culture medium.

To isolate equine MSCs from peripheral blood, 8 ml venous blood of four donors was collected from the vena jugularis using EDTA as anti-coagulans. After centrifugation at 1,000 g for 20 min at RT, the buffy coat fraction was collected and diluted 1:1 (v:v) with PBS. Subsequently, the cell suspension was gently layered on an equal volume of Percoll (density 1.080 g/ml, Sigma-Aldrich, P1644) and centrifuged for 15 min at 600 g at RT, as previously described (38). After collecting the interphase and three wash steps with PBS, cells were resuspended in 5 ml culture medium, seeded in a 25 cm^2^ culture flask and cultured at 38.5°C in a humidified atmosphere containing 5% CO_2_. Non-adherent cells were removed the following day by completely replacing the culture medium.

### Culture of MSCs

For all species, culture medium was replaced twice weekly and cells were passaged as soon as five or more well-developed colonies were observed. To this end, adherent cells were washed with PBS and incubated with 0.25% trypsin (Sigma, T4799)-−0.02% EDTA (Sigma, EDS) solution for 5 min at 38.5°C. Cold culture medium was used to block the trypsin after which the cell suspension was centrifuged for 10 min at 300 g at RT. Finally, the cell pellet was resuspended in expansion medium. Cell viability and cell numbers were assessed after staining with trypan blue (Sigma) using a Neubauer cell counter. Cells were seeded at a density of 5,000 cells/cm^2^, cultured in expansion medium and designated as passage one (P1) cells.

### Tri-lineage differentiation

After three passages, undifferentiated MSCs were induced toward the adipogenic, chondrogenic and osteogenic differentiation, respectively. Appropriate negative controls were included, being non-induced cells cultured in expansion medium. Briefly, cells for adipogenic differentiation were cultured in triplicate in 24-well plates at a density of 21,000 cells/cm^2^ in expansion medium until 90–100% confluency. Adipogenic differentiation was induced by subsequent cycles of 72 h culturing in adipogenic induction medium followed by 24 h in adipogenic maintenance medium. Adipogenic differentiation was assessed using Oil Red O (Sigma, O0625) histological staining, a lysochrome, fat-soluble dye used to stain triglycerides and lipoproteins intracellularly, with a Mayer's modified hematoxylin (Abcam, ab220365) counterstaining. Briefly, after cells were fixed using 4% paraformaldehyde (Carl Roth, 964.1, PFA), they were incubated in 60% isopropanol for 5 min and stained for 15 min with diluted Oil Red O solution (3 parts Oil Red O stock solution/2 parts dH_2_O). After two washings with dH_2_O, Mayer's modified hematoxylin was added and incubated for 2 min. Brightfield images were taken after two more wash steps.

To induce chondrogenic differentiation, 250,000 cells were centrifuged in 15 ml conical Falcon tubes for 5 min at 150 g at RT. Subsequently, 0.5 ml chondrogenic medium was added to each tube without disturbing the cell pellet. After differentiation, micromass cultures were fixed overnight with 4% PFA, pellets were embedded in 2% agarose and further processed for paraffin sectioning (slices of 5 μm). Subsequently, sections were dried at 56°C for 35 min, rehydrated using decreasing alcohol series in the Varistain Gemini slide stainer (ThermoFisher Scientific), dried for 10 min and incubated in 3% acetic acid for 3 min. Next, slides were stained for 30 min using a 1% Alcian blue (Sigma, A3157) histological staining solution (pH 2.5) to stain glycosaminoglycans (GAGs) such as hyaluronic acid in the extracellular matrix. After rinsing the slides under running tap water for 10 min, they were flushed three times with dH_2_O and placed into a 0.1% Nuclear Fast Red solution (Sigma, N3020) for 5 min. After rinsing with tap water for 1 min and one washing step with dH_2_O, slides were dehydrated using increasing alcohol series, and sections were covered with a toluene-based mounting medium (Thermo Scientific, Clearvue) and a cover glass and subsequently imaged.

Osteogenic differentiation was performed in triplicate in 24-well culture dishes with 10,000 cells/cm^2^ cultured in expansion medium until 90–100% confluency, after which osteogenic differentiation was induced. Osteogenic differentiation was evaluated after fixing the cells with 4% PFA and Alizarin Red S (Sigma, A5533) histological staining of 45 min, used to evaluate calcium deposits in the extracellular matrix, according to the manufacturer's instructions.

### Image acquisition and analysis

Images of adipogenic and osteogenic differentiation were captured using an inverted microscope (DMi1, Leica Biosystems, Nussloch, Germany) at a magnification of 100x and 200x, respectively. Images of chondrogenic differentiation were captured at 200x using a brightfield microscope (DM LB2, Leica Biosystems, Nussloch, Germany).

**Figure 1:**
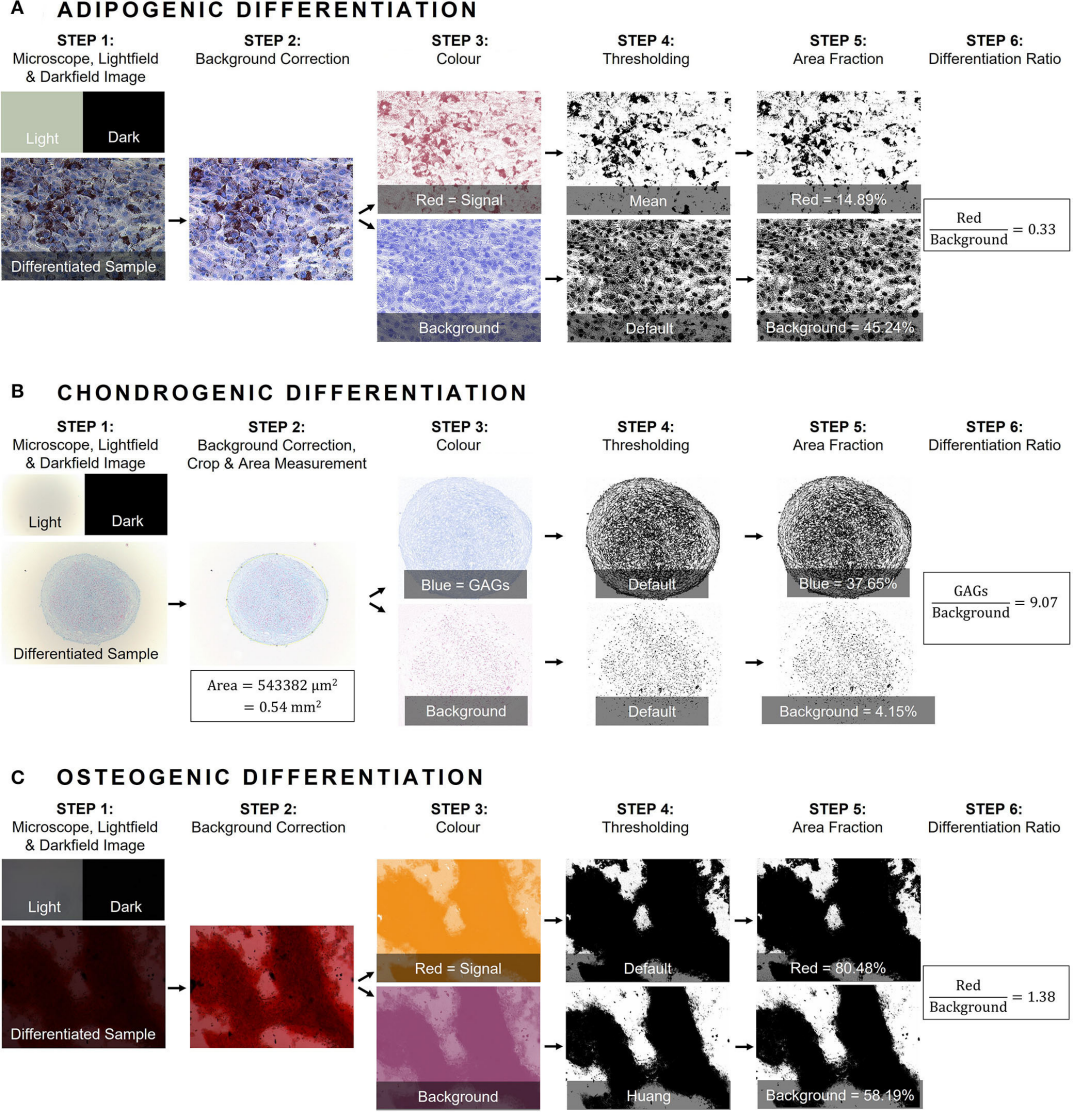
General workflow of image analysis using color deconvolution in ImageJ. **(A)** Adipogenic differentiation was confirmed with Oil Red O histological staining and Mayer's modified hematoxylin counterstaining, **(B)** Chondrogenic differentiation with Alcian Blue histological staining and Nuclear Fast Red counterstaining. Additionally, in step 2 chondrogenic pellet size (area in mm^2^) was measured, and **(C)** Osteogenic differentiation was confirmed with Alizarin Red staining. In general, a darkfield image (called “Dark”) was obtained with a closed illumination path to correct for any fixed pattern noise (step 1). Subsequently, an image of an empty well was captured as “brightfield” (called “Light”) to correct for varying background illumination (step 1). As such, images were corrected prior to image analysis for background illumination and transmittance through the well (step 2). Next, a threshold for differentiation was determined for each histological staining and separated from the second component either nuclear signal or cell culture area (step 3). Per sample, two channels were established and quantified using “*Thresholding*” (step 4). Finally, the area fraction of the differentiation channel was measured in the binarized image and divided by the area fraction of the counterpart (step 5 and 6). GAGs, glycosaminoglycans.

First, an image acquisition protocol was established to ensure consistent image acquisition and data generation. A macro was written in ImageJ macro language to analyze images in batch mode. All relevant parameters such as focus, illumination, exposure, exposure-time and white-balance were determined per image series using visual confirmation and subsequently fixed during image acquisitions. The workflow of the method for all three differentiation lineages is presented in Figure 1. ImageJ macro's were written to analyze images in batch mode and are openly available (39). With well-defined and fixed settings, a darkfield image (called “Dark”) was obtained with a closed illumination path to correct for any fixed pattern noise (hot-pixels). Subsequently, an image of an empty well was captured as “brightfield” (called “Light”) to correct for varying background illumination. As such, images were corrected prior to image analysis for background illumination and transmittance through the well, using “*ImageCalculator*” and “*Calculator_Plus”* in ImageJ to compare images between different donors and/or microscopes using following formula:


$$Corrected\;image=\frac{\left(Original-Dark\right)}{\left(Light-Dark\right)}$$

Next, three different wells per donor and four images per well were analyzed for the adipogenic and osteogenic differentiation, while three paraffin sections per donor were analyzed for the chondrogenic differentiation.

For image analysis, the method suggested by Landini et al. was applied, implementing the color deconvolution plug-in for ImageJ with user-defined values (40, 41). Per histological staining, a mixture of two different colors, related to the different stainings, are unmixed using the “*Colour Deconvolution 2”* plug-in. Each image is separated into two channels representing respective concentrations of each dye. The threshold per dye was determined by assembling an image collage of both differentiated and negative control wells of the same donor, as suggested by Landini et al. (42). Next, the obtained color-vectors were inspected visually by applying the workflow on randomly selected images of different species. If needed, the vectors were adjusted until satisfactory results were obtained. The red signal from the Oil Red O staining, identifying lipid droplets in the cytoplasm after adipogenic differentiation, is separated from the purple hematoxylin nuclear counterstaining. To evaluate chondrogenic differentiation, a blue threshold for staining GAGs in the matrix is separated from the red Nuclear Fast Red signal, used to identify the nuclei. As the chondrogenic differentiation is performed in a micromass culture system, the difference in pellet size between differentiated MSCs vs. non-induced controls was evaluated. Pellet size (area in mm^2^) was determined by fitting manually an ellipse around the pellet, after which the image was processed. Osteogenic differentiation is confirmed by identifying the calcium deposits of the osteogenic matrix using Alizarin Red S. As non-induced cells stain purple in the absence of osteogenic matrix, a threshold for the red signal is defined to identify osteogenic differentiation and is separated from the purple signal of the non-induced controls.

After determining the color vectors, channels were separated using the “*Colour Deconvolution 2”* plug-in. Per sample, two channels were established and, for mathematical operation during the analysis steps, converted into an eight-bit format. Binarization was performed using “*Thresholding”* available in ImageJ. The most appropriate auto-threshold algorithm was determined on sample images and was repeatedly visually inspected during image analyses (43). Subsequently, the area of the signal in the binarized image of each channel was measured. To calculate the differentiation ratio (DR), the area fraction of the differentiation color (being red, blue, and red for adipogenic, chondrogenic and osteogenic, respectively, channel 1) was divided by the area fraction of the second component either nuclear signal or cell culture area (channel 2) (Figure 1). In order to compare differentiation between different donors within one species, a normalized DR was obtained per donor by dividing the mean DR of differentiated wells by the mean DR of the non-induced controls.

### Statistical analysis

Data analysis was performed using R Studio (Version 1.3.1093, RStudio, PBC, Boston, MA, USA), using the package “*nlme.”* Normality was evaluated by visual examination of Q–Q plots and equality of variances was assessed by plotting the residuals against the fitted values. The effect of the condition, i.e., differentiated vs. non-induced cells, was evaluated within one species using repeated measures ANOVA (a univariate approach, condition = fixed factor and donor = random factor).

## Results

**Figure 2:**
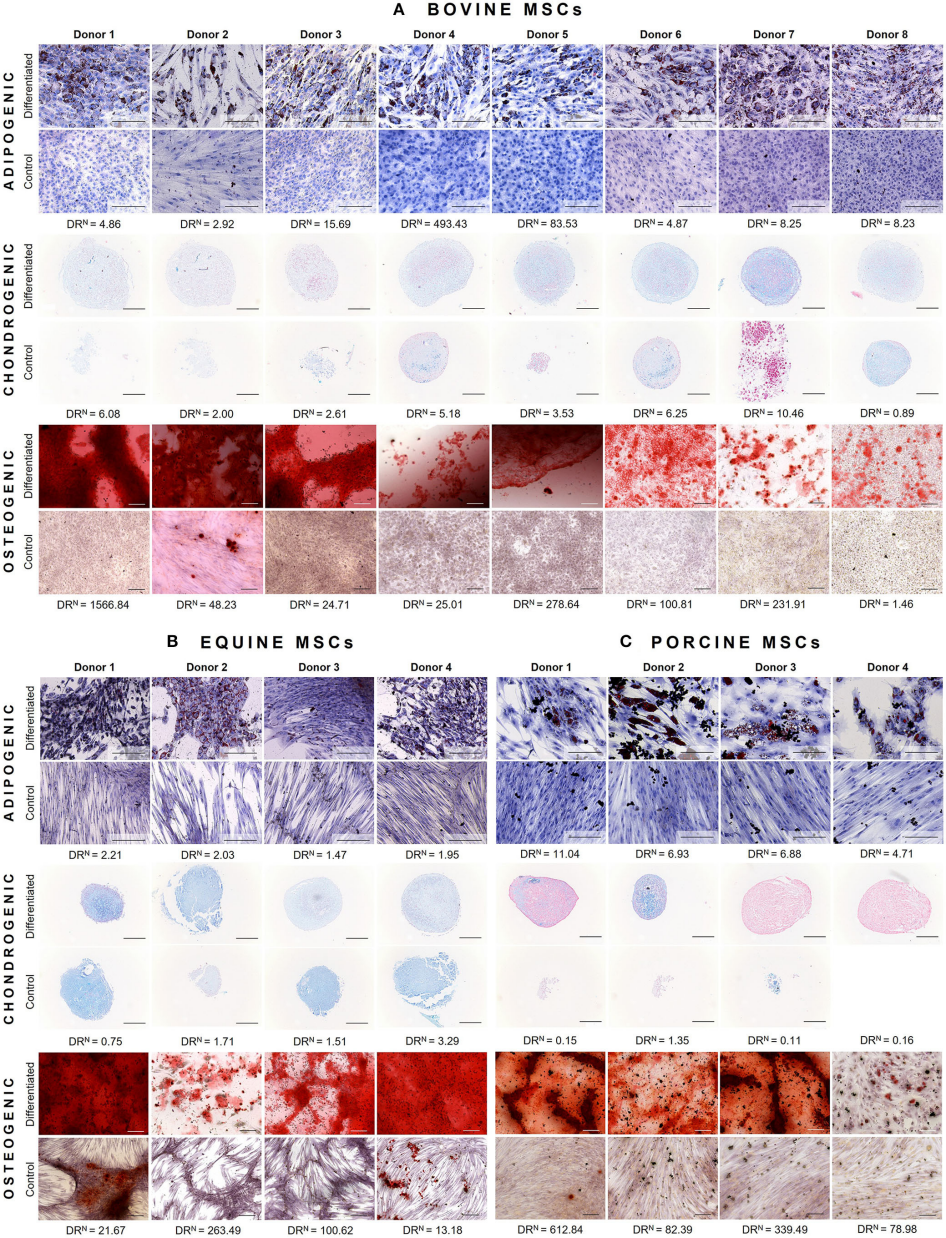
Representative images of tri-lineage differentiation potential of **(A)** bovine MSCs (*n* = 8), **(B)** equine MSCs (*n* = 4), and **(C)** porcine MSCs (*n* = 4). For each donor, both differentiated MSCs and non-induced controls are shown, as well as the normalized differentiation ratio (DR^N^). The latter is calculated by dividing the mean differentiation ratio of differentiated wells by the mean value of the non-induced controls. When this ratio is less than 1, less differentiation is observed when compared to non-induced controls; when the DR^N^ is above 1, a higher differentiation is observed. Scalebar = 200 μm.

**Figure 3:**
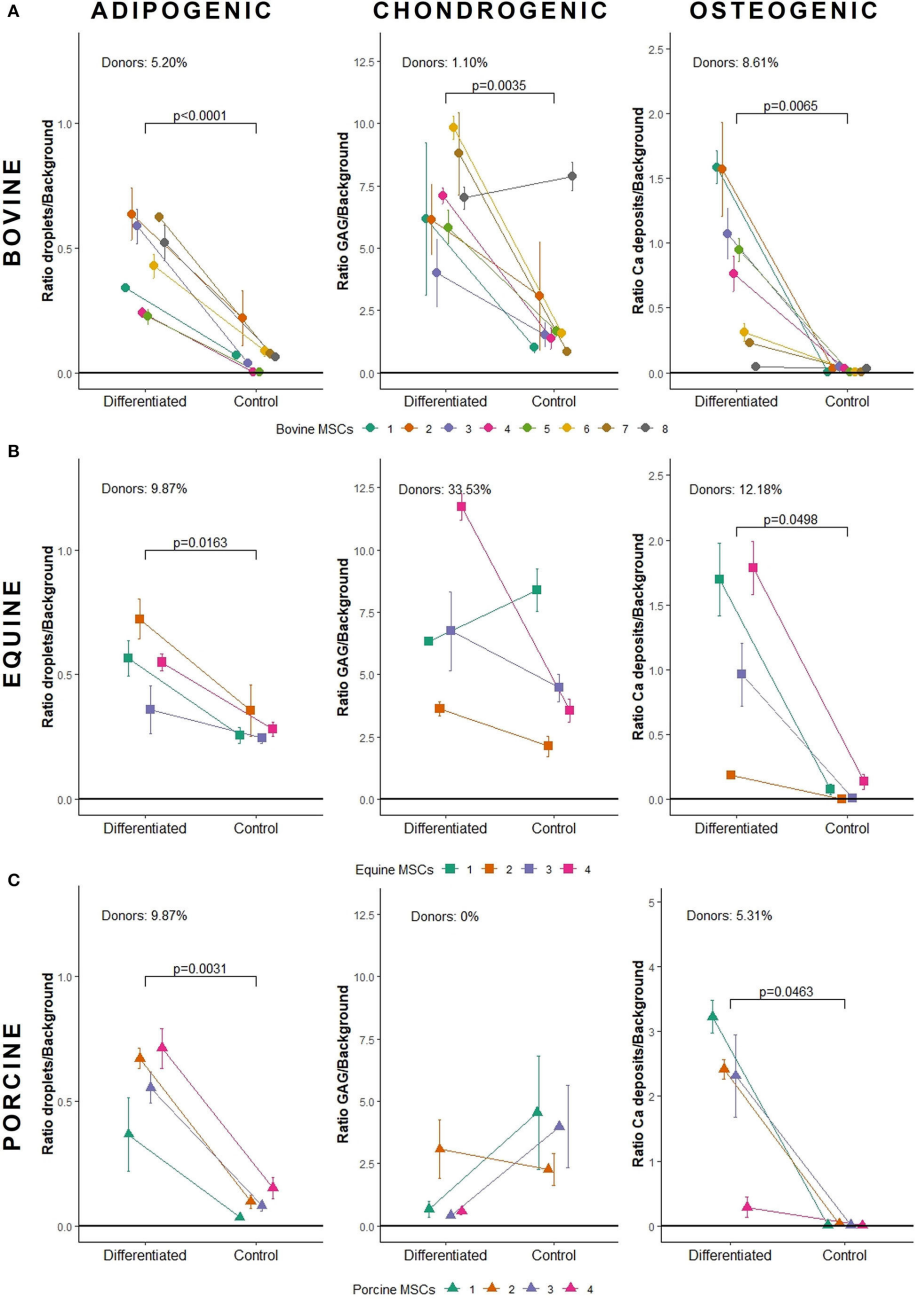
Quantification of tri-lineage differentiation potential of **(A)** bovine MSCs (*n* = 8), **(B)** equine MSCs (*n* = 4), and **(C)** porcine MSCs (*n* = 4). Donors are linked in an interaction plot and the mean differentiation ratio with standard deviation is shown per lineage. GAG, glycosaminoglycans.

**Figure 4:**
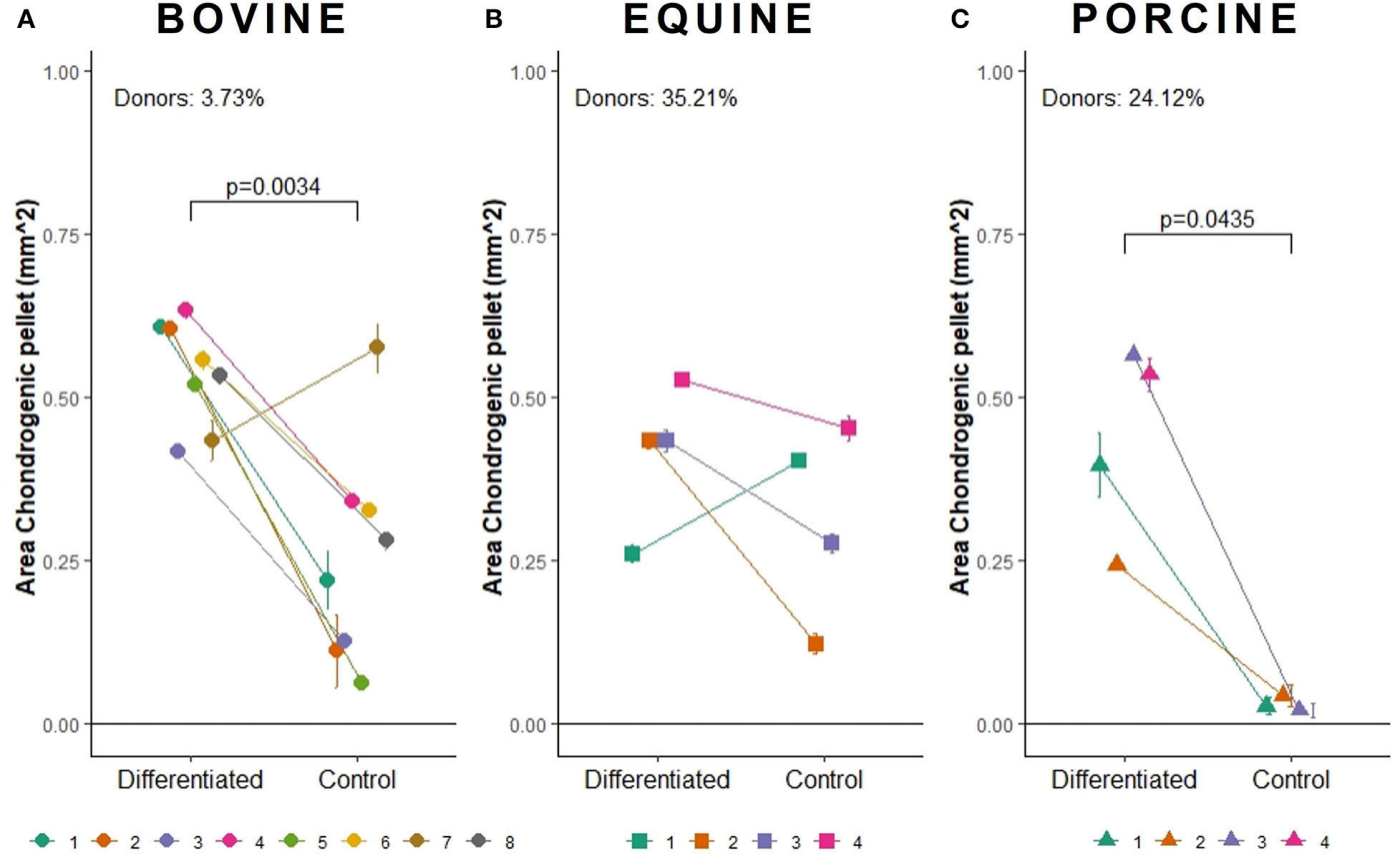
Area measurement (in mm^2^) to determine the pellet size of chondrogenically induced **(A)** bovine MSCs (*n* = 8), **(B)** equine MSCs (*n* = 4), and **(C)** porcine MSCs (*n* = 4). The mean of chondrogenic pellet area with standard deviation is shown.

**Figure 5:**
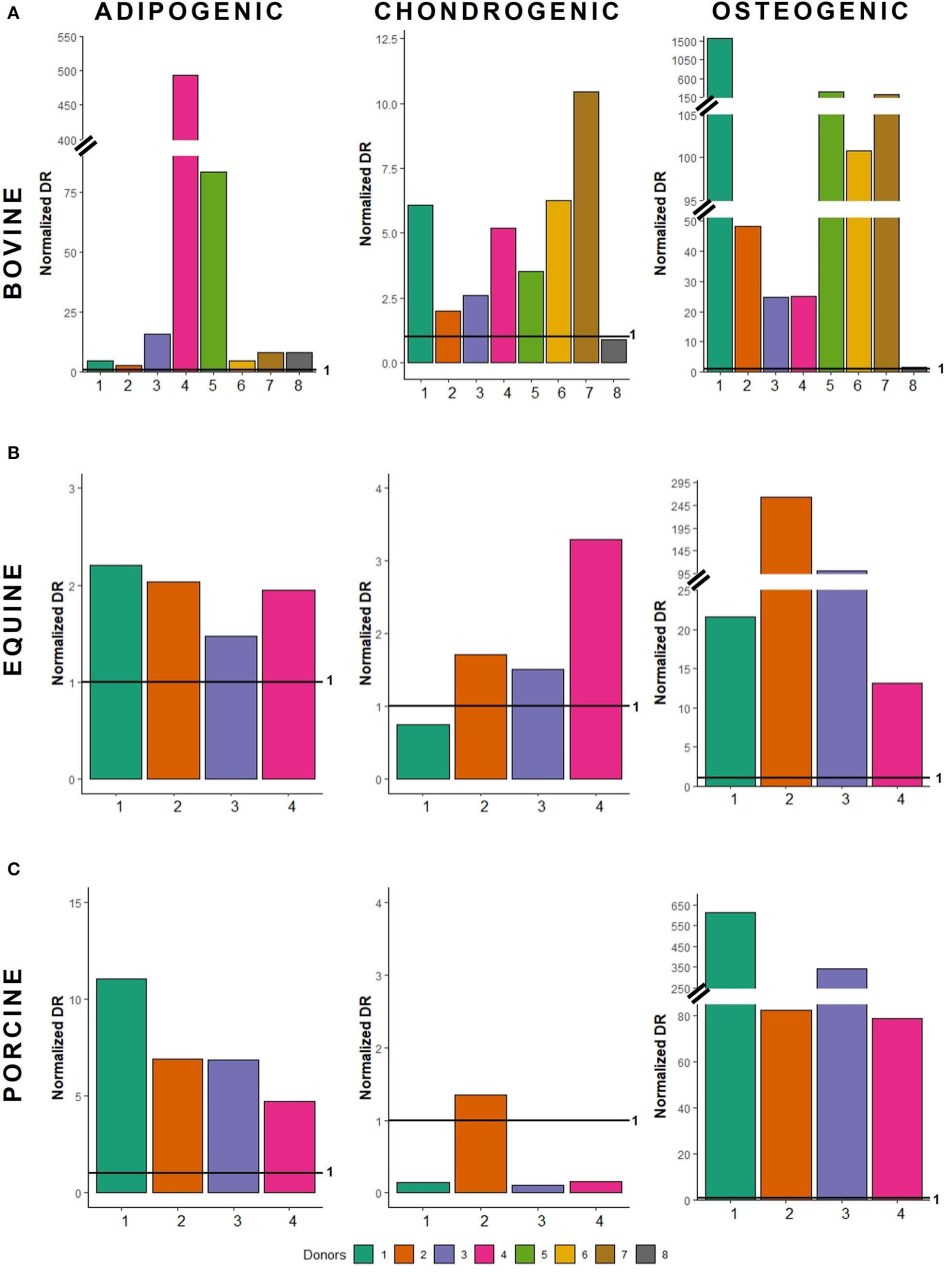
Normalized differentiation ratios of **(A)** bovine MSCs, **(B)** equine MSCs, and **(C)** porcine MSCs. For each donor, the normalized differentiation ratio (DR) is shown. The latter is calculated by dividing the mean DR of differentiated wells by the mean value of the non-induced controls. When this ratio is less than 1, less differentiation is observed when compared to non-induced controls; when the normalized DR is above 1, a higher differentiation is observed.

Bovine MSCs were isolated from eight calves and differentiated toward adipocytes, chondrocytes and osteocytes, respectively. Based on qualitative evaluation of the histological stainings, all donors were able to differentiate toward adipocytes, chondrocytes and osteocytes (Figure 2A). Clear differences in osteogenic differentiation potential were observed by qualitatively assessing the Alizarin Red S staining (Figure 2A). Quantitative evaluation indeed showed significant differences between differentiated and non-induced cells for adipogenic (*p* < 0.0001), chondrogenic (*p* = 0.0035) and osteogenic DR (*p* = 0.0065) (Figure 3A). For one donor, the chondrogenic differentiation potential was however limited when compared to non-induced controls (Figure 3A). Regarding the difference in pellet size a significant difference between chondrogenically differentiated and the non-induced pellet area was detected for bovine donors (*p* = 0.0034) (Figure 4A). Differences in differentiation potential within one donor were observed as indicated by a high standard deviation. For example, the chondrogenic DR of donor 1 and osteogenic DR of donor 2 showed a large variation (Figure 3A). Additionally, using the normalized DR, differences in differentiation potential were observed from donor-to-donor as well (Figures 2A, 5A). When the normalized DR is less than 1, less differentiation is observed in the differentiated MSCs when compared to non-induced controls; when the normalized DR is above 1, more differentiation is observed. Based on the high normalized DR, for example, donors 4 and 5 showed a superior adipogenic differentiation potential when compared to the other donors (Figures 2A, 5A). Donors 1, 5, 6, and 7, on the other hand, showed a high osteogenic potential as indicated by the high normalized DR. In contrast, donor 8 showed a very low chondrogenic and osteogenic potential, as indicated by a normalized DR of 0.86 and 1.46, respectively (Figures 2A, 5A). It is challenging to identify these differences by qualitative assessment only (Figure 2A). When taking the normalized DR into account for the chondrogenically differentiated bovine MSCs, chondrogenic differentiation of donor 8 was not confirmed as differentiated MSCs gave less differentiation signal than their non-induced counterparts, resulting in a normalized DR less than 1 (Figures 2A, 5A).

To verify whether our quantification method could be applied across species, equine and porcine MSCs were evaluated as well. Equine MSCs were isolated from four donors and all donors were able to differentiate toward adipocytes, chondrocytes and osteocytes upon qualitative assessment (Figure 2B). Again, variation in osteogenic differentiation potential was observed (Figure 2B). A higher adipogenic and osteogenic DR was observed when compared to non-induced controls (*p* = 0.0163 and *p* = 0.0498, respectively) (Figure 3B). As shown in Figure 3B, a high variation in osteogenic differentiation potential within one donor was observed as illustrated by the high standard deviation in three out of four donors. For equine donors, chondrogenic differentiation potential was limited, when comparing differentiated to non-induced samples (Figure 3B). In addition, no significant differences in pellet size were observed (Figure 4B). Regarding donor-to-donor variation, the normalized DR indicated that donors 2 and 3 were superior for osteogenic differentiation (Figures 2B, 5B). Since the non-induced controls of donors 1 and 4 showed spontaneous osteogenic differentiation, their respective normalized DR's were lower than those of donors 2 and 3 (Figures 2B, 5B), although they showed clearly osteogenic differentiation, both qualitatively in Figure 2B and quantitatively in Figure 3B. Similarly, non-induced controls of donor 1 showed spontaneous chondrogenic differentiation and the normalized chondrogenic DR of this donor is therefore less than 1 (DR^N^ = 0.75).

Porcine MSCs were isolated from four donors. Using a qualitative assessment, all donors were able to differentiate toward the three lineages (Figure 2C). Using our quantification method, a significantly increased adipogenic and osteogenic DR was observed when compared to their respective non-induced controls (*p* = 0.0031 and *p* = 0.0463, respectively). No significant difference in chondrogenic DR was observed between differentiated and non-induced controls (Figure 3C), since the normalized DR, which is calculated per donor individually, was less than 1 for all but one donors (Figures 2C, 5C). It must be mentioned that the non-induced cells of donor 4 did not form a cell pellet. Therefore, the mean DR of the non-induced controls of donors 1–3 was used to calculate the normalized DR of donor 4. Similar to our results in bovine MSCs, a significant difference in pellet size was observed between chondrogenically differentiated cells and their non-induced controls (*p* = 0.0435) (Figure 4C). Regarding variation in differentiation potential within one donor, adipogenic differentiation of donor 1 varied extensively, while chondrogenic differentiation within donor 2 varied, as well as osteogenic differentiation within donor 3 (Figure 3C). Regarding variation in differentiation potential from donor-to-donor, donor 1 showed a superior osteogenic and chondrogenic differentiation potential, when compared to the other donors. Furthermore, donor 3 showed a high osteogenic differentiation potential (Figures 2C, 5C).

## Discussion

In this study, a straightforward, simple but robust method was developed to objectively quantify tri-lineage differentiation potential of MSCs based on histological stainings routinely used to assess differentiation qualitatively. To evaluate our quantification method across species, the tri-lineage differentiation potential of bovine, equine, and porcine MSCs was assessed. Using this method, the degree of tri-lineage differentiation can be determined, regardless of microscope settings, species, histological staining, MSC source and/or isolation technique.

A major hurdle when developing MSC-based therapies is the inability to consistently produce homogeneous MSC populations. Indeed, a MSC population consists of a mixture of different stem and lineage-committed progenitor cells, also referred to as MSC heterogeneity (44). Using our quantification method, variability within one donor was observed in all species, illustrating this heterogeneity (Figure 3). Regarding donor-to-donor variation, the outcome of MSC-based therapies might improve when allogeneic MSCs of donors with, for example, known superior osteogenic differentiation potential are used to induce bone regeneration (45), such as bovine donor 1. Bovine donor 7, on the other hand, showed a superior chondrogenic differentiation potential, as illustrated by the high normalized DR (Figure 5A), and thus might be the most appropriate donor for cartilage damage repair (46). Furthermore, it should be mentioned that even subtle changes in differentiation potential can be identified using the normalized DR, such as the difference in osteogenic differentiation potential between bovine donor 4 and 5 (Figures 2A, 5A). As such, it would become possible to select the most appropriate MSC donor for each specific clinical application.

Concerning the chondrogenic differentiation potential of bovine, equine and porcine MSCs, all donors were considered to differentiate into chondrocytes using a qualitative assessment of the Alcian Blue staining. However, our quantification method showed only a limited differentiation capacity, as no significant differences between differentiated and non-induced cells were detected for equine and porcine MSCs (Figure 5). Since the chondrogenic differentiation potential was evaluated in a 3D micromass culture system, the difference in pellet size between differentiated and non-induced cells could additionally be used as parameter. When evaluating pellet size, bovine and porcine chondrogenically differentiated MSCs formed a larger pellet compared to non-induced cells. In contrast, no differences were observed between differentiated and non-induced cells for the equine MSCs. As high-density culture conditions and hypoxia in a 3D cell culture system may enable chondrogenic differentiation, the similar results of the differentiated and non-induced cells for the Alcian Blue staining of equine MSCs might be explained by spontaneous expression of GAGs (47, 48). The latter is illustrated by the large pellets formed in the non-induced equine MSCs which stained positive for Alcian Blue (Figure 2B). Similar results were observed for equine synovial membrane-derived MSCs (49). Regarding the porcine differentiated MSCs, one must keep in mind that the DR is calculated by dividing the area fraction of Alcian Blue by the nuclear signal. In the chondrogenically differentiated MSCs, the presence of glycosaminoglycans, as indicated by the Alcian blue signal, is limited while many nuclei are present. In the undifferentiated control group, no cell pellet was formed and many cells were lost during differentiation and subsequent processing of the samples, which resulted in smaller areas. As such, the DRs of chondrogenically differentiated porcine MSCs, showing some blue signal and many nuclei, are similar to those of the non-induced controls, showing little blue signal and few nuclei. These findings result in normalized DRs close to 1. Furthermore, porcine chondrogenic differentiation in a 3D system is not reported as far as we know, and chondrogenic differentiation of porcine MSCs in general is frequently lacking (34, 50). Further optimizing the chondrogenic differentiation medium per species might be indicated to achieve optimal chrondrogenic differentiation. Further improvement might be achieved by changing the composition and/or concentration of growth factors, such as TGF-β and insulin-like growth factor, supplemented to the medium (51–53).

Prior to bringing new MSC therapies to the market, authorities such as the European Medicines Agency and the U.S. Food and Drug Administration require that they are tested in both a small and large animal model. Based on the similarities of porcine physiology with human, the pig is considered as a very relevant animal model for myocardial infarction, osteochondral defects, wound healing, etc. (54). Our method can support the selection of the most appropriate porcine MSC donor in pre-clinical studies in which the pig is used as model for human. The horse, on the other hand, represents an interesting animal model for preclinical orthopedic research (55). In equine regenerative medicine, MSCs are frequently used to treat osteoarthritis and tendon lesions (56). Identifying specific donors with improved chondrogenic or tenogenic capacities may therefore improve the outcome of clinical studies by providing more consistent results. Both equine and human regenerative medicine would benefit from this approach.

In addition to regenerative applications, undifferentiated MSCs are often used for their anti-inflammatory and immune-modulating capacities (57). Some studies, however, reported that the immune-modulating capacities of MSCs might be impacted by differentiation (58, 59). For instance, chondrogenically differentiated MSCs lost their immune-suppressive capacities and became immunogenic (59). In that context, it is important to verify the absence of spontaneous MSC differentiation, especially when undifferentiated MSCs are used as an immune-suppressive therapy. In addition, adipose tissue-derived MSCs might be contaminated with pre-adipocytes and mature adipocytes, which are potentially immunogenic (17). Both examples illustrate another application of the quantification method reported in this study, namely, to verify whether undesirable cell phenotypes are present when using undifferentiated MSCs therapeutically.

Finally, this quantification method is not limited to evaluate the tri-lineage differentiation potential of MSCs, but it can be applied to other cell types, tissues or staining dyes. With minor modifications, for example, neurogenic differentiation of MSCs can be quantified by detecting neuronal Nissl bodies using a cresyl violet staining (60). Our method is based on color deconvolution (61), in which a brightfield image is decomposed into two separate channels, one for the staining of interest (per differentiation) and one for the counterpart (42, 62). This color deconvolution-based approach can easily be extended to a third staining component.

Nevertheless, there are some limitations to consider when using this method. Color deconvolution is based on eight-bit images and requires a preliminary definition of the color deconvolution components, which can be challenging and as such, is prone to errors (15, 42). It is essential to perform a background correction to provide a neutral color background and a uniformly illuminated field. Furthermore, it must be mentioned that some dyes scatter rather than absorb light or are neutral (gray), and thus cannot be unmixed by color deconvolution. Additionally, when using color deconvolution on fluorometric or immunohistochemical images, precise protein concentrations cannot be determined as these are non-stoichiometric reactions (15, 61, 63). As for all image analysis methods, results depend on the quality of the stained tissue section, as well as on the properties of the images which are affected by many factors, such as tissue fixation method, histological tissue processing, sectioning, staining procedures and image acquisition (62, 64). Finally, it might be interesting to compare the (normalized) DR with other established quantification techniques, such as absorbance assays, in future research.

In conclusion, an efficient method based on color deconvolution was developed in this study to quantify tri-lineage differentiation potential of MSCs across species. We were able to demonstrate differences in differentiation potential within one donor and between donors. Using our well-defined and open access method, the degree of tri-lineage differentiation can be determined in future MSC research. The knowledge gained using this method represents an important asset for regenerative medicine applications as it will support the development of new, more targeted MSC-therapies and improve the consistency and quality of commercially available MSC products.

## Data availability statement

Data presented in this study are available upon request from the corresponding author. Image J macro's are available via https://github.com/MeeremansMarguerite/MSC-Differentiation-Quantification.git.

## Author contributions

EH was involved in conception and design, sample collection, differentiation experiments, and manuscript writing. MM was involved in creating the ImageJ macro's, data analysis, conception and design, and manuscript writing. BD was involved in data analysis and manuscript writing. MO was involved in manuscript writing. KC was involved in histological staining and data acquisition. CDS was involved in conception and design, data analysis, and manuscript writing. All authors contributed to the article and approved the submitted version.

## Funding

This research was partly funded by the Flanders Research Foundation (FWO), SBO grant number S002821N (CustoMeat). MM is funded by the FWO, SB grant number 1S02822N.

## Conflict of interest

The authors declare that the research was conducted in the absence of any commercial or financial relationships that could be construed as a potential conflict of interest.

## Publisher's note

All claims expressed in this article are solely those of the authors and do not necessarily represent those of their affiliated organizations, or those of the publisher, the editors and the reviewers. Any product that may be evaluated in this article, or claim that may be made by its manufacturer, is not guaranteed or endorsed by the publisher.
